# Changes in the Chemical Components of Processed Rehmanniae Radix Distillate during Different Steaming Times

**DOI:** 10.1155/2022/3382333

**Published:** 2022-02-17

**Authors:** Shujuan Xue, Yu Fu, Xiaoya Sun, Suiqing Chen

**Affiliations:** School of Pharmacy, Henan University of Chinese Medicine, Zhengzhou, Henan, China

## Abstract

Distillate was obtained in different processing cycles of processed Rehmanniae Radix (PRR). In this study, we investigated the chemical compositions of distillates 1 (Dis1) to 9 (Dis9) via GC-MS and LC-MS. Differences between Dis1–Dis9 were noticeable. A total of 13 and 21 compounds were detected via GC-MS and LC-MS, respectively, including organic acids, furans, alcohols, iridoid glycosides, phenylpropanoid glycosides, and saccharides. The relative contents of compound 2,5-hydroxymethylfurfural and furans all gradually increased with steaming time. Other compounds, however, exhibited a negative trend or fluctuated. Of these compounds, iridoid glycosides and phenylpropanoid glycosides were unstable and easily degraded, which led to a gradually decreasing concentration with increased steaming times. In addition, the degradation products were mainly derived from catalpol and acteoside, among which catalpol mainly existed as aglycone and its rearranged products. However, acteoside was converted into verbasoside through the removal of caffeoyl. Some volatile alcohols, such as phenylethyl alcohol, hydroxyphenyl ethanol, and 3-hydroxy-4-methoxybenzoic acid, were also likely from the degradation of acteoside and its homologs. These results provide an important reference basis for the processing methods, quality evaluation, and rational clinical application of PRR and its distillate.

## 1. Introduction

Rehmanniae Radix (RR), the root of *Rehmannia glutinosa* Libosch., is one of the four major “huaiyao” in the Henan Province and has been widely used for the treatment of various diseases in East Asia for thousands of years [1]. Three types of Rehmanniae Radix are widely used as a common Chinese Medicine owing to different processing methods, namely, fresh Rehmanniae Radix (FRR), raw Rehmanniae Radix (RRR), and processed Rehmanniae Radix (PRR). PRR is a product of RR obtained by steaming the dried root with rice wine followed by sun-drying for nine cycles, and in doing so not only could that change the character and taste, according to the quality standard of “black as paint, sweet as wheat,” but it also enhanced the effect of nourishing yin and reinforcing the kidney [2]. It is believed to be efficient in enriching yin and reinforcing the kidney in Traditional Chinese Medicine (TCM), prevent osteoporosis [3, 4], possess renoprotective activity [5, 6], and prevent cardiovascular diseases [7]. It has become one of the most popular dietary supplements for healthcare [8].

The components of PRR have been analysed using different techniques. The main compositions of PRR include phenethyl alcohol glycosides [9], iridoid glycosides [10], ionone glycosides [11], and saccharides [12, 13], the concentration of which will differ depending on the processing time and temperature.

Most distillate would be produced due to cooling of water vapour condensation. Water-soluble compounds could be distilled with steam, and therefore, the distillate appeared to be black and thick and gave off a sweet and sour smell. The distillate was a byproduct of PRR and generally used as supplementary material mixed with PRR of different processing times. There were different compositions in different processing times. To date, the chemical components in the distillate of PRR have not yet been thoroughly characterised. Majority of previously published studies on the chemical constituents mainly focused on PRR [14]. Some literature studies have reported that the main components of PRR, such as 5-hydroxymethylfurfural (5-HMF), catalpol, sucrose, maltose, and glucose, varied depending on the number of steaming and drying cycles. It was proposed that six cycles or more were an appropriate PRR process [15]. However, some studies suggested that PRR steamed and dried nine times can normalize an increased blood lipid content and reduce the number of free radicals increased by hyperlipidemia. But whether the distillate has the same components and clinical effect as PRR requires further study.

To the best of our knowledge, studies on the chemical compositions of PRR distillate have been limited to some saccharides despite the great diversity of compounds contributing to the distillate development and utilisation. Our lab detected many saccharides of distillate of PRR at different steam times, including monosaccharides and oligosaccharides. The chemical profile demonstrated that the saccharides gradually changed from Dis2 to Dis8, indicating that the processing cycle has a larger impact on the distillate [12]. Only saccharide components have been determined, however, and further study is needed for other components. Due to significant variations in the chemical components under different processing times, further study of how distillate changes could provide more comprehensive and scientific evidence for the development and utilisation of distillate.

Distillate, formed by water vapour under high temperature, includes many volatile components and water-soluble constituents. Volatile components are usually detected via gas chromatography-mass spectrometry (GC-MS), which is widely used in various industries, such as food [16] and agriculture [17]. However, water-soluble constituents are mostly polar compounds, which can be detected via high-performance liquid chromatography with mass spectrometry (LC-MS).

Here, a method combining GC-MS and LC-MS was developed for the simultaneous determination of PRR distillate with different steam times. The result is an accurate and simple assay for a more comprehensive component analysis of PRR distillate. Furthermore, the results could reveal the principle by which constituents change and compare the distillate chemical profiles to reveal the relationship between chemical components and processing technology.

## 2. Materials and Methods

### 2.1. Chemicals and Reagents

**Figure 1:**
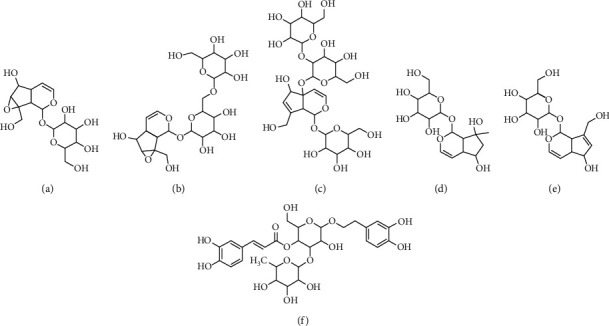
The chemical structure of standards: (a) catalpol, (b) rehmannioside A, (c) rehmannioside D, (d) leonuride, (e) aucubin, and (f) verbascoside.

The following standards for iridoid glycosides and phenylpropanoid glycosides were purchased from APTBio (Shanghai, China): catalpol, rehmannioside A, rehmannioside D, leonuride, aucubin, and verbascoside, n-alkanes (C8–C20; Sigma-Aldrich, USA). The structures of these standards are shown in Figure 1. Methanol, acetonitrile, and formic acid (HPLC grade) were supplied by Fisher Scientific (Fairlawn, NJ, USA). Ethyl acetate and petroleum ether (HPLC grade) were purchased from Shanghai Chemical Reagent Co. (Shanghai, China). Water was purified by a Milli-Q system (Millipore, Milford, MA, USA).

### 2.2. PRR Distillate

Dis1 to Dis9 were collected during the nine processing cycles of PRR, respectively. The liquid formed when water vapour cooled in nine cycles. Three samples were collected during each processing cycle, among which, the volume of each samples was about 1000 mL, and a total of 27 samples were collected during February–May 2020 and stored at 4°C prior to analysis.

### 2.3. Preparation of Sample and Standard Solutions

Dis1–Dis9 (10 mL) were extracted by 30 mL ethyl acetate three times. The distillate from each wash was merged. Then, each extract was concentrated to dryness using a rotary evaporator and the residue was dissolved in 5 mL ethyl acetate. After dehydration by sodium sulfate anhydrous, the solution was filtered through a 0.2 *μ*m Millipore filter and then stored at −20°C prior to the GC-MS analysis.

All samples were diluted 10 times with distilled water and then centrifuged at 12,000 rpm for 10 min. The supernatants were stored at −20°C prior to UHPLC-LTQ-Orbitrap-MS/MS analysis.

A standard stock solution of each compound with a concentration of 0.5 mg/mL was separately prepared using distilled water. All solutions were stored at 4°C prior to use.

### 2.4. UHPLC-LTQ-Orbitrap-MS/MS Analysis

The chromatographic separation was performed on a Dionex UltiMate 3000 UHPLC system (Thermo Scientific, Germering, Bavaria, Germany). The samples were injected onto a Waters HSS T3 column (Waters Corp., Milford, MA, USA) (1.7 *μ*m, 2.1 × 100 mm) operated at 35°C. The separation of the samples was achieved using a gradient elution consisting of water containing 0.1% formic acid (A) and acetonitrile (B) at a flow rate of 0.3 mL/min as follows: 17% B within 0–5 min, 17%–20% B within 5–15 min, 20%–23% B within 15–20 min, 23%–24% B within 20–25 min, 24%–17% B within 25–30 min, and 17% B within 30–32 min. The UV detection wavelength was at 254 nm and the injection volume was 1 *μ*L.

The results of mass spectrometry were obtained via electrospray ionisation (ESI) in the positive ion mode with a mass range of 80–1000 Da. The ESI source parameters were set as follows: ion spray voltage, 4.2 kV; capillary temperature, 350°C; capillary voltage, 23 V; tube lens voltage, 90 V; and sheath (N2) and auxiliary gas (He) flow rates, 25 and 3 arbitrary units, respectively.

### 2.5. GC-MS Analysis

Gas chromatography analysis was conducted using a Thermo Fisher TSQ 8000 gas chromatograph–mass spectrometer. The WD-5 MS silica capillary column (30 m × 0.25 mm i.d., 0.25-*μ*m film thickness) was used to analyse nonpolar constituents. The optimised GC programme was as follows: initial temperature of 45°C and held for 15 min, then ramped to 60°C at a rate of 2°C/min and held at 60°C for 5 min, and finally increased at 3°C/min to 300°C and held at this temperature for 3 min. Helium (99.99% purity) was used as carrier gas with a flow rate of 1.0 mL/min. The injector temperature was 280°C; split ratio, 20 : 1; and injection volume, 1 *μ*L. The WD-170 MS silica capillary column (30 m × 0.25 mm i.d., 0.25 *μ*m film thickness) was used to analyse polar constituents. The optimised GC programme was as follows: initial temperature of 45°C and held for 5 min, then ramped to 200°C at a rate of 2°C/min and held at 190°C for 2 min, and finally increased at 20°C/min to 230°C and held at this temperature for 10 min. Helium (99.99% purity) was used as carrier gas with a flow rate of 1.0 mL/min. The injector temperature was 250°C; split ratio, 30 : 1, and injection volume, 1 *μ*L.

The MS conditions were as follows: the electron impact energy of the mass-selective detector was 70 eV and full-scan range was 50–500 amu. FFAs were identified by mass spectra database (NIST14 Library) and the Kovats retention index for each aroma compound was calculated with a homologous series of n-alkanes (C8–C20) under the same GC-MS conditions.

### 2.6. Data Preprocessing

In this study, the XCalibur 3.0 software was used for mass spectrum analysis (Thermo Fisher, Waltham, MA, USA). Principal component analysis (PCA) was conducted to evaluate the changes in the chemical profiles of Dis1 to Dis9 using the SIMCA-P 14.0 software (Umetrics AB, Sweden). The relative quantitative data of compounds showing significant differences were obtained with different methods; among them, the nonvolatiles were relatively quantified by area normalization method and the volatiles were relatively quantified on ion peak intensities. One-way analysis of variance (ANOVA) of these quantitative results was conducted using IBM SPSS Statistics 20.0 (SPSS Institute, Chicago, IL, USA). GraphPad Prism 8.0 (GraphPad Software Inc., USA) was used to create the charts of the results.

## 3. Results and Discussion

### 3.1. Chemical Profiles of Dis1 to Dis9 by LC-MS

**Figure 2:**
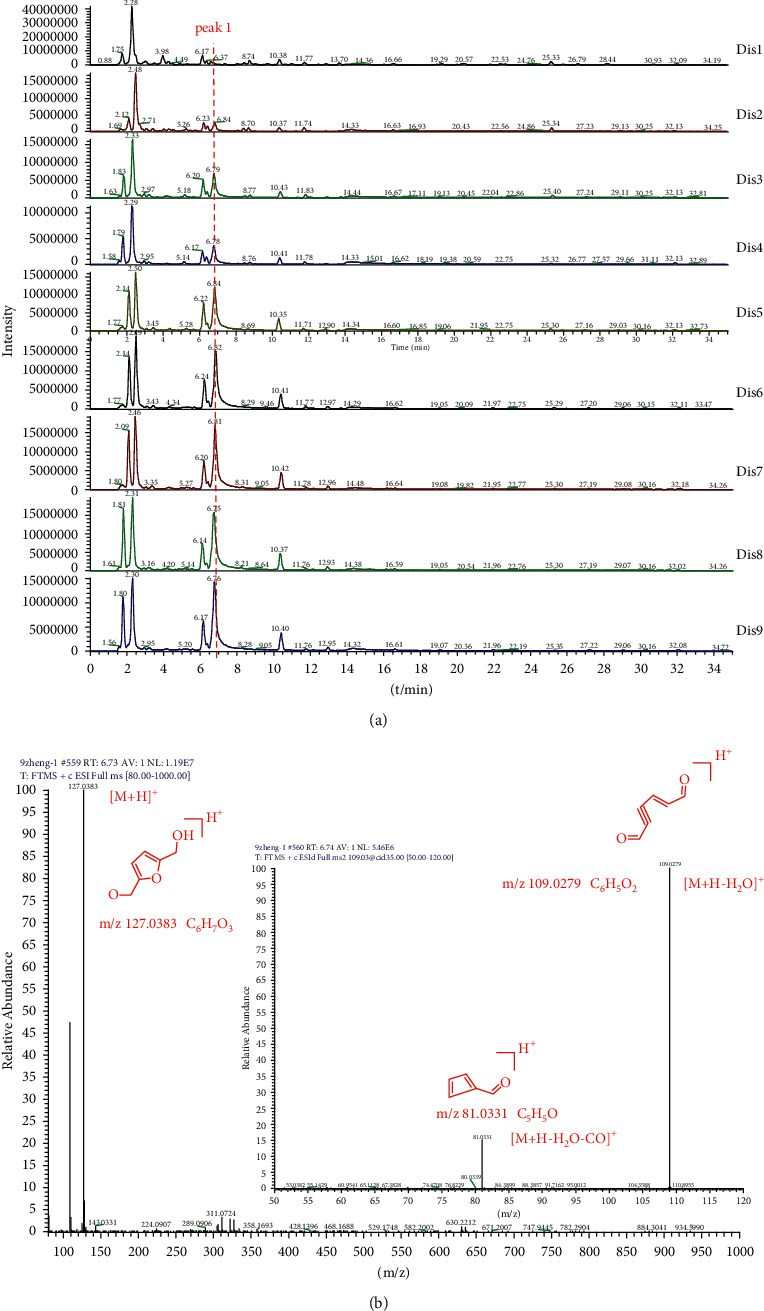
Total ion chromatograms (TICs) (a) of Dis1 to Dis9 and the CID MSn spectrum (b) of 5-hydroxymethylfurfural.

According to the LC-MS analysis, the chemical profiles were detected in PRR distillate. The total ion flow diagram is presented in Figure 2(a). As can be seen from Figure 2(a), with the increase in steaming times, the number of peaks has no significant change, but the content, especially peak 1, exhibits an increasing trend. Subsequently, we found that peak 1 gave rise to a [M + H]^+^ ion at *m*/*z* 127.0383 (C_6_H_7_O_3_). From the CID MSn spectrum (Figure 2(b)), the precursor ion at *m*/*z* 127.0383 (C_6_H_7_O_3_) produced a [M + H-H_2_O]^+^ ion at 109.0279 (C_6_H_5_O_2_) through ring opening and the loss of H_2_O. Then, the fragment ion at *m*/*z* 109.0279 (C_6_H_5_O_2_) further produced a [M + H-H_2_O-CO]^+^ ion at 81.0331 (C_5_H_5_O) through the loss of CO. According to the mass spectrum analysis, we speculated that the compound is 5-HMF. Two reaction mechanisms have been proposed for 5-HMF conversion, namely, the fructose and 3-deoxyglucosone (3-DG) mechanisms [18]. In the fructose mechanism, glucose firstly isomerises to fructose, then to fructose dehydrate, and finally to 5-HMF via a fructose intermediate. However, the 3-DG mechanism directly dehydrates glucose to HMF via a 3-DG intermediate [19]. From the reaction mechanisms, we can determine that 5-HMF was mainly converted by glucose and fructose, which is closely related to the glucose or fructose content in the samples. Furthermore, the formation rate of 5-HMF from glucose is slower than that from fructose due to the different ring structures. Consequently, the fructose mechanism may play a significant role in the 5-HMF conversion process. In our previous studies, we found that the concentration of fructose was highest in Dis1, then decreased, and then gradually increased after Dis4. This trend prompts the hypothesis that 5-HMF is mainly converted by the fructose mechanism.

**Figure 3:**
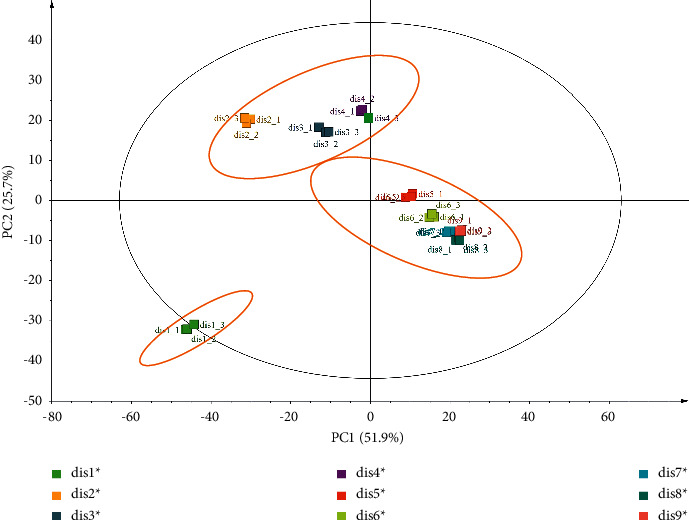
PCA score of Dis1 to Dis9.

To compare the difference between Dis1 and Dis9, unsupervised PCA was conducted. PCA is a common statistical method that uses the idea of reducing dimensions to turn multiple variables (indicators) into a few unrelated comprehensive variables. It is used in the comprehensive evaluation of TCM. In this section, PCA was employed to discriminate the Dis1 from the Dis9 samples and to screen the characteristic components of the different distillate times. The PCA results are presented in Figure 3. PCA revealed that principal components 1 and 2 in the distillate data processing expressed 51.9% and 25.7% of the variables in Dis1 to Dis9, suggesting that the two principal components could be utilised to comprehensively evaluate the quality of distillate. In addition, we can clearly see that the samples were grouped into three categories. Dis1 was separately classified into one category, whereas Dis2–Dis4 and Dis5–Dis9 were divided into two other categories. The classification results are consistent with the saccharides of our previous studies [12]. Dis1 was formed by direct steaming for more than 20 h after soaking RRR. The concentration of the compounds in Dis1 was relatively low because RRR contained a lot of water, and therefore, it is farther away from the other samples on the PCA diagram. In Dis2 to Dis4, the chemical compositions, such as oligosaccharides, polysaccharides, and iridoid glycosides, would be hydrolysed or degraded with the increase in the steaming times, and therefore, their chemical profiles are similar. However, for Dis5 to Dis9, the hydrolysis and degradation of chemical compositions may have been balanced. After a long steaming, the Maillard reaction occurred due to the large formation of reducing monosaccharide-fructose. Meanwhile, 5-HMF is Maillard reaction product, whose content increased gradually. All the above descriptions indicated that the chemical composition of distillate changed in quality or quantity during the different steaming processes, resulting in the samples being divided into three categories.

### 3.2. Identification of Chemical Components in Dis1 to Dis9 by LC-MS

**Figure 4:**
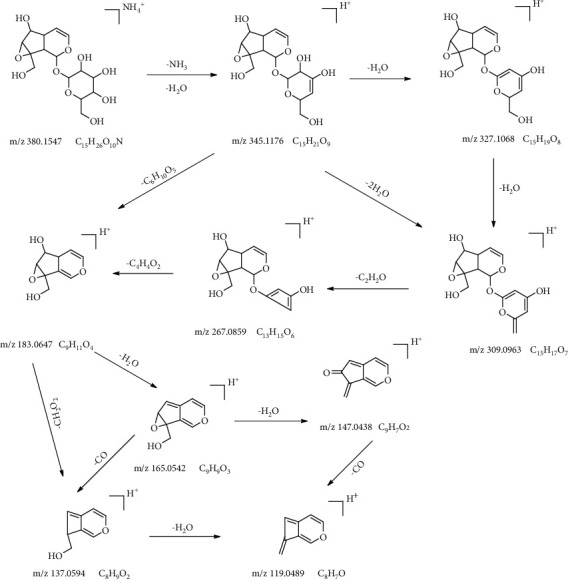
The fragmentation pathways in the positive ion mode of catalpol.

**Figure 5:**
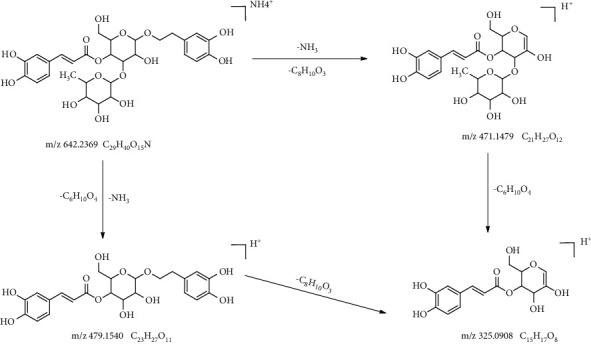
The fragmentation pathways in the positive ion mode of acteoside.

To identify the chemical components in Dis1 to Dis9, a UHPLC system coupled with an LTQ-Orbitrap-MS was used for the chemical profiling of PRR distillate. The accurate mass number of the compound was first calculated according to its molecular formula using the XCalibur 3.2 software, and then, the chemical components in Dis1 to Dis9 were confirmed by the required retention times, accurate masses, MS/MS spectra fragments, and comparison of commercially available standards. A total of 21 peaks were identified, including 9 prototype components and 12 of their degradation products. Among them, five compounds originated from catalpol, four compounds originated from acteoside, and two other compounds originated from leonuride and geniposide, respectively. Their mass spectrometry data are presented in detail in Table 1. Five of the nine prototype components were identified by comparison of those standards, namely, catalpol, rehmannioside D, rehmannioside A, leonuride and acteoside. The five identified compounds were iridoid glycosides and phenylpropanoid glycosides, for which the possible fragmentation pathways are presented in Figures 4 and 5. Iridoid glycosides were the most important active constituents of RR as its mother nucleus is iridoid alcohol and has a hemiacetal and cyclopentane ring structure. It mainly exists in plants in the form of C1-OH and glycosides. Catalpol and leonuride are lignan glycosides, but rehmannioside A and rehmannioside D are diglycosides and triglycosides, respectively. Taking the reference substance of catalpol as an example, under the positive ion mode, a molecular ion peak at *m*/*z* 380.1536 [M + NH_4_]^+^ of catalpol was produced, then a molecular ion peak at *m*/*z* 380.1536 separately produced [M-H_2_O]^+^, [M-2H_2_O]^+^, and [M-3H_2_O]^+^ ions at *m*/*z* 345.1176, 327.1068, and 309.0963, respectively, through a continuous loss of H_2_O. A fragment ion *m*/*z* 309.0963 further lost C_2_H_2_O and C_4_H_4_O to form *m*/*z* 183.0647 [M-C_6_H_12_O_6_]^+^ fragment ion. The fragmentation pathway indicated that catalpol first lost the functional groups on the mother nucleus and then the mother nucleus produced [M-C_6_H_12_O_6_-H_2_O]^+^ and [M-C_6_H_12_O_6_-2H_2_O]^+^ ions at *m*/*z* 165.0542 and 147.0438 by the loss of H_2_O. Finally, the cyclopentane ring lost a CO when it was cracked. From the above ion fragments, it can be seen that the mass spectral fragmentation pattern of iridoid glycosides was to first lose the functional groups, then fracture the mother nucleus structure through a series of H_2_O losses, and finally produce characteristic fragment ions that lose 18 Da (H_2_O), 36 Da (2H_2_O), 126 Da (C_6_H_6_O_3_), and 28 Da (CO). Similarly, rehmannioside D, rehmannioside A, and leonuride have the same fragmentation patterns. According to the retention time of catalpol standards and MS/MS spectra fragmentations, compound 4 was identified as catalpol. In addition, compounds 7, 8, and 9 were, respectively, identified as rehmannioside D, rehmannioside A, and leonuride.

Meanwhile, other iridoid glycosides were confirmed through the above cracking rules. Compound 1 presented a molecular ion at *m*/*z* 382.1691 [M + NH_4_]^+^, after which the MS/MS spectrum demonstrated that it produced a fragment ion at *m*/*z* 185.0800. After the loss of 126 (Da), it produced fragment ions at *m*/*z* 167.0695 and 149.0591. All were 2 (Da) more than the characteristic fragment ions of catalpol, and thus, we tentatively identified them as dihydrocatalpol. Compound 2 gave rise to an [M + H]^+^ ion at *m*/*z* 201.0748. It has also the same fragment ion as catalpol and accurate masses were 179 (Da) less than it. This result indicated that compound 2 was formed due to the loss of a hexose from compound 1. It is presumed that compound 2 may be iridoid. Similarly, compound 10 was identified as leonuride aglycone according to the identical fragmentation pattern.

Phenylpropanoid glycosides were also important active ingredients of RR, including acteoside, isoacteoside, and echinacoside. Among them, acteoside was generally used as the indices for the evaluation of the RR quality. Taking the reference substance of acteoside as an example to analyse the decomposition regularity, the fragmentation pathways are presented in Figure 5. Acteoside exhibited a molecular ion at *m*/*z* 642.2369 [M + NH_4_]^+^. The precursor ion at *m*/*z* 642.2369 [M + NH_4_]^+^ produced an *m*/*z* 471.1479 ion through the loss of C_8_H_10_O_3_. Losing the fragment ions equivalent to the structure of hydroxytyrosol, the fragment ion at *m*/*z* 471.1479 has the same structure as cistanoside F. We defined them as compounds 13 and 12. Then, the fragment ion at *m*/*z* 471.1479 could be further fragmented to produce the ion 325.0908. In addition, the precursor ion at *m*/*z* 642.2369 [M + NH_4_]^+^ can also first produce ion at *m*/*z* 479.1536 through the loss of C_6_H_10_O_4_, followed by the loss of C_8_H_10_O_3_, to generate *m*/*z* 325.0908. According to the above cracking rules, compound 18 was identified as isoacteoside for having the same accurate masses and MS/MS fragmentation as acteoside. Compound 14 was identified as echinacoside, according to the [M + NH_4_]^+^ ion at *m*/*z* 804.2881, literature data, and MS/MS fragmentation.

### 3.3. Analysis of the Degradation Products of the Prototype Components by LC-MS

A total of 12 PRR degradation products were identified in Dis1 to Dis9. Their prototype components mainly included catalpol, acteoside, and leonuride. The results indicated that catalpol, acteoside, and leonuride can degrade during the steaming process. There are mainly the aglycone of catalpol and leonurus in the distillate, which is formed by the loss of glycosyl through acid hydrolysis. These glycosides were not stable under acidic conditions and easily further degraded. The aglycone of catalpol converted to 1,5-dialdehyde under high temperature and acidity. Meanwhile, 1,5-dialdehyde can also further polymerise to form the polymers. It has been reported that the polymers further loose water and form a macromolecular compound with conjugated structures, which leads to the significant darkening of the distillate. The polymers may have had a macromolecular structure that excluded it from distillation, as we did not detect it. Furthermore, the hydroxyl of some aglycone is esterified with the acid to form lipid degradation products.

**Figure 6:**
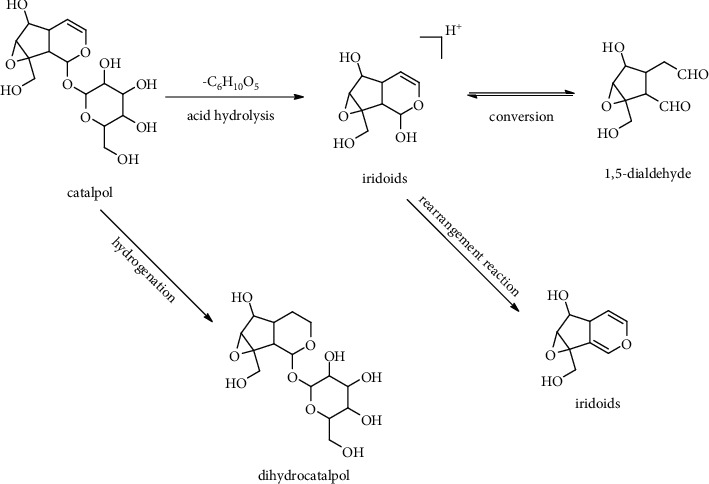
The degradation pattern in the positive ion mode of catalpol.

**Figure 7:**
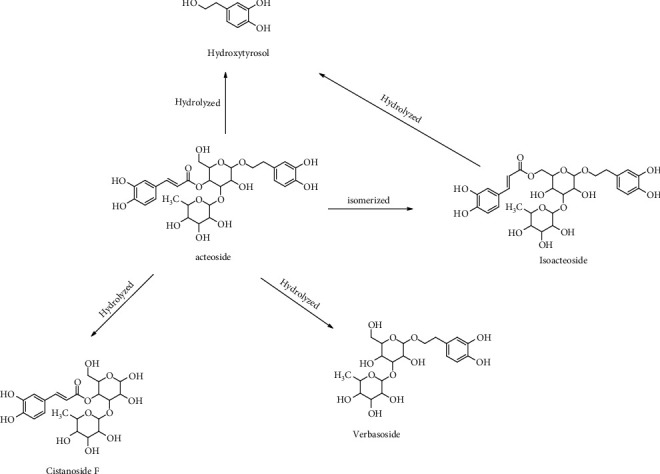
The degradation pattern in the positive ion mode of acteoside.

Different from the degradation of catalpol, acteoside was mainly isomerised to isoacteoside and then hydrolysed to cistanoside F, hydroxytyrosol, and verbasoside. Interestingly, the degradation pattern of acteoside was similar to its MS fragmentation pathways. The possible degradation pattern is presented in Figures 6 and 7.

### 3.4. Analysis of the Chemical Components in Dis1 to Dis9 by LC-MS

**Figure 8:**
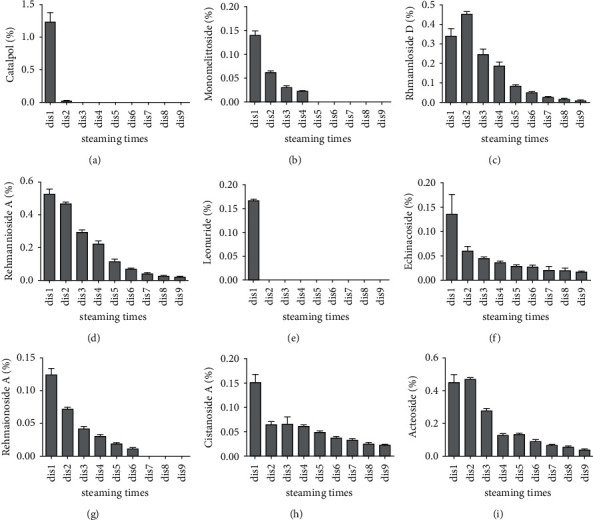
The relative contents of 9 prototype components in Dis1–Dis9.

**Figure 9:**
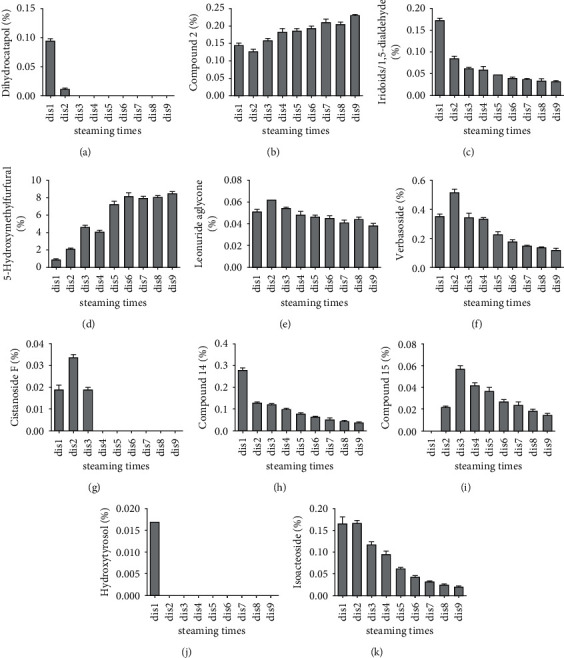
The relative contents of 12 degradation products in Dis1–Dis9.

In this section, the relative contents of 21 identified components were expressed as mean value ± standard deviation of triplicate determinations and presented in a chart (Figures 8 and 9). The quantitative results were obtained via area normalization. Among the nine prototype components, the relative content of 5-HMF gradually decreased with the steaming times, from 0.912% ± 0.045% in Dis1 to 8.501% ± 0.13% in Dis9. 5-HMF was a dehydration decomposition product of glucose or fructose under acidic conditions. Some reports indicated that glucose first isomerises to fructose, followed by fructose dehydration to 5-HMF via fructose intermediate or direct dehydration of glucose to HMF through a 3-DG intermediate. The formation rate of 5-HMF from glucose is slower than that from fructose [19]. However, the formation of 5-HMF was closely related to glucose and fructose. In our previous research, some saccharide compounds were determined and analysed. The results indicated that the fructose content was significantly altered by different processing cycles. It was highest in Dis1, then decreased, and again gradually increased after Dis4. However, glucose was lowest in Dis1 and then increased and remained relatively stable. These findings indicate that fructose may be first converted to 5-HMF in the Maillard reaction due to the higher content in the first four steaming cycles. Then, the fructose contents gradually increased with the decrease in the rate of the Maillard reaction. This result also corresponds to the slower formation rate of glucose.

With regard to the other prototype components, their relative contents all exhibited a decreasing tendency. Leonuride was only found in Dis1, catalpol only existed in Dis1 to Dis2, and monomelittoside was detected in Dis1 to Dis4. Their concentrations reached 0.168% ± 0.002%, 1.244% ± 0.132%, and 0.142% ± 0.008% in Dis1, respectively. Furthermore, rehmannioside D and rehmannioside A were found in Dis1 to Dis9. The reason may be that they all belong to the iridoid glycosides, of which rehmannioside D and rehmannioside A are polyglycosides with relatively stable properties, whereas leonuride, catalpol, and monomelittoside are monoglycosides and prone to degradation. Likewise, acteoside, cistanoside A, and echinacoside have existed in nine processing cycles. Their degradation rates were relatively slower owing to their macromolecular structure.

In addition to the prototype components, the relative contents of 12 degradation products identically showed a certain change in principle. Like 5-HMF, the relative content of compound 2 also gradually increased from 0.143% ± 0.008% to 0.231% ± 0.002% during the entire steaming process. Compound 2 was a product of catalpol by structural rearrangement of aglycone, indicating that catalpol mainly degraded to glycogen and was rearranged in the processing system. Certainly, other degradation products were produced, such as compound 14, dihydrocatalpol, and iridoids. Similarly, four degradation products of acteoside were found, namely, verbasoside, cistanoside F, isoacteoside, and hydroxytyrosol. Among them, verbasoside was the highest, reaching 0.52% in Dis2 and then gradually decreasing, indicating that acteoside may be mainly degraded to verbasoside by removing caffeoyl after processing. It has been reported in the literature that the stability of acteoside is poor and could significantly decrease under the conditions of light and alkalinity [20, 21]. Other studies have demonstrated that acteoside is mainly hydrolysed by verbasoside under high temperature, which was consistent with our results. In addition, acteoside could also isomerise to form isoacteoside and be simultaneously degraded gradually with steaming times. Certainly, according to the complexity of the degradation reaction, other degradation products have been found, such as compound 15. It was preliminarily inferred by the esterification reaction of geniposide and formic acid, but its concentration was lower, only 0.01%–0.05%.

### 3.5. Determination of Volatile Constituents in Dis1 to Dis9 by GC-MS

**Figure 10:**
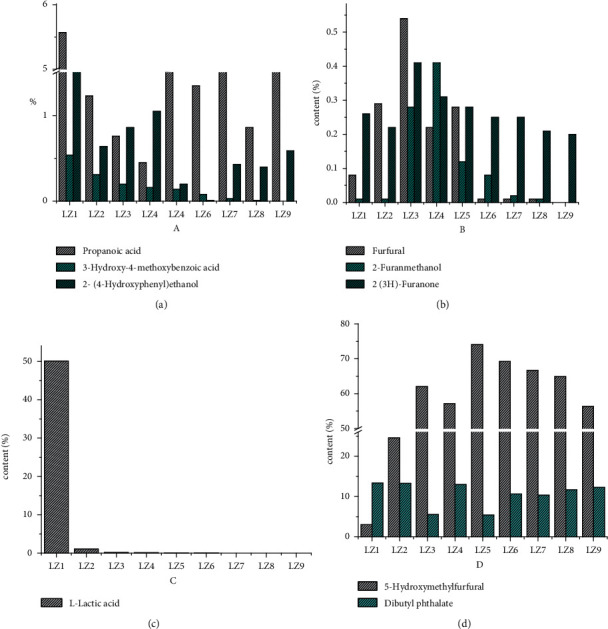
Percentage contents of each compound in Dis1–Dis9.

To further study the volatile constituents in the PRR distillate, Dis1–Dis9 were analysed via GC-MS. To improve the accuracy of identification, identification of the peaks was performed by searching the National Institute of Standards and Technology (NIST) 14. MS library (a minimum match quality of 85% was used as the criterion). The RIs were compared with NIST 14. MS library (the difference between the measured value and theoretical value was less than 20). The GC-MS total ion chromatograms of Dis1–Dis9 are presented in the supplementary materials (Figure 10). The constituents in Dis1–Dis9 were obviously different from each other. The identified compounds and their relative percentage concentrations (%) are presented in Table 2. A total of 13 compounds were identified. Among these distillations, Dis1 contains more compounds, mainly including organic acids, furans, and alcohols. The concentration of L-lactic acid is highest in Dis1, where it exceeds 50%. The percentage content of each compound in Dis1–Dis9 is presented in Figure 2. In steaming, the total content of organic acids decreased, whereas the total content of furans increased. For organic acid, L-lactic acid exhibited a significant reduction from 50% to 1.09% in Dis1 and Dis2 and then gradually decreased in Dis3–Dis9. In Dis9, L-lactic acid could not be detected. Propanoic acid and 3-hydroxy-4-methoxybenzoic acid were highest in Dis3 and then gradually decreased in Dis4–Dis9. Furans are another main constituent in Dis1–Dis9. Among these furans, the content of 5-HMF significantly increased from Dis1 to Dis3 and then remained relatively stable. The dynamic change in the 2-furanmethanol and 2(3H)-furanone content showed a “˄” shape and the content of these two constituents was highest in Dis3 and Dis4.

Combined with the results from the LC-MS analysis, a large concentration of 5-HMF was observed in the GC-MS sample. Some furans, such as furanone, furfural, and furanmethanol, were also identified in the sample. Literature [22] reports that furans could be synthesised by fructose or glucose dehydration under acidic or heating conditions. Therefore, with the increase in the steaming time, total furan content increased. Moreover, some volatile alcohols were identified in the sample. In the LC-MS analysis, acteoside could be hydrolysed to alcohols. Therefore, we inferred that some volatile alcohols, such as phenylethyl alcohol, hydroxyphenyl ethanol, and 3-hydroxy-4-methoxybenzoic acid, were probably from the degradation of acteoside and its homologs.

## 4. Conclusions

Many saccharides were detected in our previous research. In this study, we coupled GC-MS with UHPLC-LTQ-Orbitrap-MS/MS to identify degradation products in the PRR distillate of different steam times. A total of 13 compounds were identified via GC-MS, mainly organic acids, furans, and alcohols. Twenty-one compounds were characterised through LC-MS, containing 9 prototype components and 12 degradation products, namely, iridoid glycosides and phenylpropanoid glycosides. The results of the GC-MS and LC-MS indicated that the constituents in Dis1–Dis9 were different compounds. Most components had a higher content in the first few steaming cycles, especially in Dis1. Further processing gradually decreased or showed a peak followed by a decreasing trend. The degradation pattern demonstrated that the 12 degradation products were mainly derived from catalpol and acteoside, among which catalpol mainly existed as aglycone and its rearranged products. However, acteoside was converted into verbasoside through the removal of caffeoyl. Our results indicated that the chemical composition of distillates was similar to PRR and has a great development and utilisation value. However, we have to point out that our results were not perfect; there are some deficiencies. Unfortunately, we did not quantify accurately these prototype components and degradation products, only explaining the variation and transformation mechanisms. But these problems would be solved in further research. We would explore further the dose-response relationship between compounds and pharmacological activities. Not withstanding their limitation, our research results provide an important reference basis for processing methods, quality evaluation, and rational clinical application of PRR and its distillate.

## Figures and Tables

**Table 1:** Quantitative analysis results of chemical components in Dis1–Dis9 by LC-MS.

Compound no.	Identification	*t*_*R*_ (min)	Formula	*m*/*z*	ppm	MS2	M+	Steaming times	Source
1	Dihydrocatalpol	2.47	C_15_H_24_O_10_	382.1691	−4.376	185.0804 [M-C_6_H_12_O_6_-H_2_O]^+^ 167.0699 [M-C_6_H_12_O_6_-2H_2_O]+	[M + NH_4_]^+^	Dis1-Dis2	Catalpol
2	Compound 2	2.56	C_9_H_8_O_3_	182.0803	−4.996	96.0439, 87.0436	[M + NH_4_]^+^	Dis1–Dis9	Catalpol
3	Iridoids	3.53	C_9_H_13_O_5_	201.0748	−4.874	183.0643 [M-H_2_O]^+^ 165.0538 [M-2H2O]+	[M + H]^+^	Dis1–Dis9	Catalpol
4	1,5-Dialdehyde								
5	Catalpol	3.91	C_15_H_22_O_10_	380.1536	−4.268	183.0643 [M-C_6_H_12_O_6_]^+^ 165.0538 [M-C_6_H_12_O_6_-H_2_O]^+^	[M + NH_4_]^+^	Dis1-Dis2	Prototype components
6	5-Hydroxymethylfurfural	6.73	C_6_H_7_O_3_	127.0383	−4.358	109.0279 [M-H_2_O]^+^ 81.0331 [M-H_2_O-CO]^+^	[M + H]^+^	Dis1–Dis9	Saccharides
7	Monomelittoside	7.36	C_15_H_22_O_10_	380.1536	−3.926	327.1071 [M-2H_2_O]^+^ 165.0542 [M-C_6_H_12_O_6_-H_2_O]^+^	[M + NH_4_]^+^	Dis1–Dis4	Prototype components
8	Rehmannioside D	8.40	C_27_H_42_O_20_	704.2578	−4.202	489.1582 [M-C_6_H_12_O_6_]^+^ 471.1472 [M-C_6_H_12_O_6_-H_2_O]^+^	[M + NH_4_]^+^	Dis1–Dis9	Prototype components
9	Rehmannioside A	8.70	C_21_H_32_O_15_	542.2057	−4.160	345.1170 [M-C_6_H_12_O_6_]^+^ 183.0648 [M-2C_6_H_12_O_6_]^+^	[M + NH_4_]^+^	Dis1–Dis9	Prototype components
10	Leonuride	9.76	C_15_H_24_O_9_	366.1745	−3.790	169.0851 [M-C_6_H_12_O_6_]^+^ 151.0746 [M-C_6_H_12_O_6_-H_2_O]^+^	—	Dis1	Prototype components
11	Leonuride aglycone	11.35	C_9_H_13_O_3_	169.0853	−5.682	151.0746 [M-H_2_O]^+^ 141.0539 [M-C_2_H_4_]^+^	[M + H]^+^	Dis1–Dis9	Leonuride
12	Verbasoside	11.77	C_20_H_30_O_12_	480.2054	−4.585	317.1219 [M-C_6_H_10_O_4_]^+^ 309.1169 [M-C_8_H_9_O_3_]^+^	[M + NH_4_]^+^	Dis1–Dis9	Acteoside
13	Cistanoside F	13.06	C_21_H_28_O_13_	506.1847	−4.141	—	—	Dis1–Dis3	Acteoside
14	Compound 14	13.69	C_9_H_15_O_5_N	217.0959	3.720	171.0909 [M-CH_2_O_2_]^+^ 144.0802 [M-CH_2_O_2_-C_2_H_2_]^+^	[M + NH_4_]^+^	Dis1–Dis9	Catalpol
15	Compound 15	14.89	C_12_H_13_O_5_	237.0748	−4.218	191.0694 [M-CH_2_O_2_]^+^ 163.0382 [M-C_3_H_6_O_2_]+	[M + H]^+^	Dis2–Dis9	Geniposide
16	Hydroxytyrosol	15.85	C_8_H_11_O_3_	155.0695	−4.228	—	—	Dis1	Acteoside
17	Echinacoside	20.57	C_35_H_46_O_20_	804.2881	−4.873	625.2086 [M-C_6_H_10_O_5_]^+^ 471.1472 [M-C_6_H_10_O_5_-C_8_H_9_O_3_]^+^	[M + NH_4_]^+^	Dis1–Dis9	Prototype components
18	Rehmaionoside A	22.53	C_19_H_34_O_8_	408.2574	−4.271	211.1684 [M-C_6_H_12_O_6_]^+^ 193.1579 [M-C_6_H_12_O_6_-H_2_O]^+^	[M + H]^+^	Dis1–Dis6	Prototype components
19	Cistanoside A	22.79	C_36_H_48_O_20_	818.3037	−4.899	639.2257 [M-C_6_H_10_O_5_]^+^	—	Dis1–is9	Prototype components
20	Acteoside	25.26	C_29_H_36_O_15_	642.2363	−4.633	479.1527 [M-C_6_H_10_O_3_]^+^ 325.0905 [M-C_14_H_20_O_6_]^+^	[M + NH_4_]^+^	Dis1–Dis9	Prototype components
21	Isoacteoside	26.70	C_29_H_36_O_15_	642.2380	−1.877	479.1527 [M-C_6_H_10_O_3_]^+^ 325.0905 [M-C_14_H_20_O_6_]^+^	[M + NH_4_]^+^	Dis1–Dis9	Acteoside

**Table 2:** Volatile constituents and content in Dis1–Dis9 by GC-MS.

No	*t*_*R*_	Compounds	MW	Molecular formula	RI (measured values/theoretical value)	Different steaming times (contents)
1	5.51	Propanoic acid	118	C_5_H_10_O_3_	810/815	1 (5.57%), 4 (0.45%), 5 (1.51%), 6 (1.35%), 7 (2.1%), 8 (0.86%), 9 (2.26%)
2	6.65	Furfural	96	C_5_H_4_O_2_	832/833	1 (0.08%), 2 (0.29%), 3 (0.54%), 4 (0.22%), 5 (0.28%), 6 (0.01%), 7 (0.01%), 8 (0.01%), 9 (—)
3	7.76	2-Furanmethanol	98	C_5_H_6_O_2_	853/859	1 (0.01%), 2 (0.01%), 3 (0.28%), 4 (0.41%), 5 (0.12%), 6 (0.08%), 7 (0.02%), 8 (0.01%), 9 (—)
4	30.69	2-Acetylpyrrole	109	C_6_H_7_NO	1086/1064	1 (0.02%),
5	32.78	Phenylethyl alcohol	122	C_8_H_10_O	1086/1116	1 (0.44%)
6	37.49	2(3H)-Furanone	102	C_4_H_6_O_3_	1167/1169	1 (0.26%), 2 (0.22%), 3 (0.41%), 4 (0.31%), 5 (0.28%), 6 (0.25%), 7 (0.25%), 8 (0.21%), 9 (0.2%)
7	38.42	4H-Pyran-4-one	142	C_6_H_6_O_4_	1186/1196	1 (0.23%)
8	41.30	5-Hydroxymethylfurfural	126	C_6_H_6_O_3_	1228/1233	1 (3.02%), 2 (24.58%), 3 (62.04%), 4 (57.13%), 5 (74.11%), 6 (69.26%), 7 (66.7%), 8 (64.9%). 9 (56.35%)
9	45.08	Acetic acid	166	C_9_H_10_O_3_	1296/	1 (0.88%), 2 (0.57%), 3 (0.55%), 4 (0.86%), 5 (0.26%), 6 (0.17%), 7 (0.01%), 8 (—), 9 (0.04%)
10	50.99	2-(4-Hydroxyphenyl)ethanol	138	C_8_H_10_O_2_	1426/1434	1 (2.21%), 2 (0.64%), 3 (0.86%), 4 (1.05%), 5 (0.2%), 6 (0.01%), 7 (0.43%), 8 (0.4%), 9 (0.59%)
11	56.74	3-Hydroxy-4-methoxybenzoic acid	168	C_8_H_8_O_4_	1560/	1 (0.54%), 3 (0.2%), 4 (0.16%), 5 (0.14%), 6 (0.08%), 7 (0.03%), 8 (0.01%), 9 (—)
12	70.76	Dibutyl phthalate	278	C_16_H_22_O_4_	1959/1965	1 (13.35%), 2 (13.28%), 3 (5.56%), 4 (13.01%), 5 (5.43%), 6 (10.62%), 7 (10.35%), 8 (11.7%), 9 (12.29%)
13	26.49	L-Lactic acid	90	C_3_H_6_O_3_	840/838	1 (50.09%), 2 (1.09%), 3 (0.17%), 4 (0.12%), 5 (0.05%), 6 (0.06%), 7 (—), 8 (—), 9 (—)

## Data Availability

The data used to support the findings of this study are available from the corresponding author upon request.
